# Prevalence, Awareness, Treatment, Control, and the Associated Factors of Diabetes in an Iranian Kurdish Population

**DOI:** 10.1155/2019/5869206

**Published:** 2019-09-03

**Authors:** Roya Safari-Faramani, Fatemeh Rajati, Kamran Tavakol, Behrooz Hamzeh, Yahya Pasdar, Mehdi Moradinazar, Farid Najafi

**Affiliations:** ^1^Department of Epidemiology, Research Center for Environmental Determinants of Health, School of Public Health, Kermanshah University of Medical Sciences, Kermanshah, Iran; ^2^Department of Health Promotion, Research Center for Environmental Determinants of Health, School of Public Health, Kermanshah University of Medical Sciences, Kermanshah, Iran; ^3^School of Medicine, Howard University, Washington, DC, USA; ^4^Department of Nutrition, Research Center for Environmental Determinants of Health, School of Public Health, Kermanshah University of Medical Sciences, Kermanshah, Iran

## Abstract

**Aims:**

This study is aimed at estimating the prevalence, awareness, treatment, and glycemic control of diabetes mellitus (DM) and its associated factors in an Iranian Kurdish population.

**Methods:**

Baseline data of the Ravansar Non-communicable Disease (RaNCD) cohort study, consisting of adults aged 35-65 years, were used. Diabetes was defined as a fasting plasma glucose (FPG) of 126 mg/dl or higher, being on diabetes medication, and/or diabetes confirmed by a health practitioner.

**Results:**

Nine thousand nine hundred ninety-nine participants were assigned to this study. The prevalence of DM, awareness, treatment, and glycemic control of DM were 8.19, 74.97, 74.75, and 32.68, respectively. Based on the adjusted models, increased age (*p* < 0.01); obesity or overweight (*p* < 0.01); being ex-smoker (*p* < 0.05); suffering from dyslipidemia (*p* < 0.01), hypertension (*p* < 0.01), or both of them (*p* < 0.01); and positive family history in the first-degree relatives (*p* < 0.01) were strongly associated with a high risk of DM, while engagement in regular physical activity (*p* < 0.05) was a protective factor. Female gender (*p* < 0.01), being older than 55 years, positive family history in the first-degree relatives (*p* < 0.01), suffering from both hypertension and dyslipidemia (*p* < 0.01), and obesity or overweight (*p* < 0.005) were negatively associated with DM awareness. Being married and widowed (*p* < 0.05 and <0.05) and a high BMI (*p* < 0.01) were strong predictors of receiving treatment for DM. Six to nine years of schooling (*p* < 0.05) and suffering from hypertension (*p* < 0.05) increased the probability of DM being controlled.

**Conclusions:**

When the prevalence of DM is notable, awareness and probability of receiving treatment and controlling FPG are of particular importance. A considerable proportion of the patients were aware and on treatment, which may partly be due to improving primary health care services in Iran.

## 1. Introduction

Diabetes mellitus (DM) is one of the main health challenges worldwide. According to recent reports, the prevalence of DM in adults is on the rise. Currently, around 6.4% of adults are suffering from DM, and it is estimated to rise to 7.7% by 2030 mainly due to increasing burden of its related risk factors [1]. Unfortunately, a considerable proportion of affected populations are not aware of the DM status.

In Iran, the morbidity, mortality, awareness, and management of DM are matters of concern. Based on the latest result of a nationwide survey conducted in 2011, around 11.4% of Iranian adults are suffering from DM. According to that study, the prevalence of DM increased by 35% in comparison to that in 2005 [2]. It is projected that the number of diabetic patients in Iran will probably rise to ten million by 2030 [3]. Furthermore, the prevalence of DM risk factors has risen considerably [4]. Results of repeated surveys of noncommunicable disease risk factors revealed that more than half of Iranian adults aged 45-64 years had at least three risk factors for DM such as smoking, overweight or obesity, eating inadequate fruits and vegetables, hypertension, and low physical activity [2].

Regarding the silent nature of DM, a substantial proportion of diabetic cases are unaware of their status. A previous study demonstrated that retinopathy, chronic kidney disease, coronary heart disease, and neuropathy were diagnosed in 22%-36% of DM patients ten years after the initial diagnosis [5]. Patients' self-management is the key to diabetic control and management [6]. To manage and prevent the DM complications, heightened awareness of the disease status is a major prerequisite, which may result in a better control of FPG and slowing the disease progression and the onset of its complications [7]. The proportion of DM awareness worldwide varies from 10.8% in Angolans to 87.1% in Portuguese patients with DM [8, 9].

In Iran, approximately 76% of diabetic patients were reportedly aware of their DM status [2]. This rate varies considerably across the country and over time. Yazdanpanah et al. [10] reported that only less than half of diabetic patients in Ahvaz (southwest of Iran) were aware of their DM status. Another study conducted in Isfahan (central part of Iran) demonstrated that approximately 54% of diabetic patients were aware of their disease, with only less than half receiving treatments [11].

The time gap between the patients' awareness and their health status is essential for the efficacy of treatments. Bridging this gap requires estimating its magnitude in different populations and the related predictors. To our knowledge, there is scarce evidence on the awareness, treatment, and particularly control of DM in Iranian patients. Although literature on the prevalence of diabetes is rich in Iran, updated estimation on the awareness and control of the disease in different subgroups is very limited. We conducted “Ravansar Non-communicable Disease (RaNCD)” as the first prospective cohort study established in a large western Iranian Kurdish population. The main purpose of this study was to investigate the prevalence, awareness, treatment, control, and the related determinants of DM in this subgroup of the Iranian population at large.

## 2. Materials and Methods

For the purpose of this study, we used the baseline data of RaNCD, one of the substudies of the national Prospective Epidemiological Research Studies in IrAN (PERSIAN) [12] conducted in Ravansar as one of the western cities of Kermanshah Province. Eligible participants were adults aged 35-65 years who were permanent residents of urban or rural areas of Ravansar. In addition, they were willing to participate in the study. They had good general health status and were able to communicate. The study protocol has been described in detail elsewhere [13].

### 2.1. Study Population

A total of 10065 participants was enrolled, covering approximately 75% of the eligible individuals out of 13472 residents in the area. The baseline phase of this study was completed over three years (2014-2017). Trained staff visited and interviewed potential participants at their houses and encouraged them to enroll in the study. The day before the interviewers, the staff phoned the participants, reminded them of their appointment time, and asked them to bring along their medical records and medications to the interview.

Informed consent forms designed for the study were including the aim, procedures, and participants' rights. All participants signed the informed consent forms promptly before the interview.

### 2.2. Procedures

Related data were collected during the interview followed by anthropometric and biochemical assessments by clinical practitioners and healthcare workers whom were trained specifically for this study. Demographic and socioeconomic data were collected during the interviews at the healthcare clinic in Ravansar devoted to this study. The anthropometric and biochemical assessments were also performed by trained personnel. The participants' body mass indices (BMI) were grouped into three categories of underweight (≤18.5), normal (18.5-24.9), and overweight/obese (≥25) [14].

Weight and height (with 0.1 cm accuracy) were measured using a Bio Impedance Analyzer BIA (InBody 770 BIOSPACE, Korea) and a stadiometer, respectively. Blood pressure was measured using a manual sphygmomanometer.

Central obesity was defined by waist to hip ratio (WHR) values of ≥0.90 and ≥0.85 for men and women, respectively [15]. The 24-hour physical activity measured sports, work, and leisure time activities on an average weekday and was grouped into three categories of low (24-36.5), moderate (36.6-44.9), and vigorous (≥45) METs (metabolic equivalent of task) [13]. Dyslipidemia was considered as having an LDL cholesterol of ≥160 mg/dl, a total cholesterol of ≥240 mg/dl, HDL cholesterol of <40 mg/dl, triglycerides of ≥200 mg/dl, or a history of taking medications for dyslipidemia [16].

A Persian adapted food frequency questionnaire was used to assess the dietary status of the participants. According to the WHO recommendations, a daily healthy diet is defined as consuming at least 400 g fruits and vegetables, less than 30% of total energy intake of total fat, and less than 10% of total energy of free sugar and consuming less than 5 g salt per day [17].

The wealth index as a measure of socioeconomic status (SES) was defined based on the asset data including home ownership, area per capita, and room per capita, having a freezer, laundry machine, dish washer, personal computer, access to internet, motorcycle, car (based on its price), vacuum cleaner, and TV at the household, and having a cellphone, computer, laptop, access to internet, and car (based on its price) for personal use. The total asset score was calculated via principal component analysis (PCA) applied on the asset data, which is the sum of scores for each asset variable. Based on this score, participants were ranked and then divided into five quintiles including poorest, second poor, middle, second rich, and richest [18].

Blood pressure (BP) was measured following a 10-minute rest in sitting position. Both arms were measured twice with the cuff size adjusted to the arm circumference. Four BP measurements were taken to determine the mean systolic and diastolic pressures (SBP and DBP). Those with a SBP ≥ 140 mmHg and/or DBP ≥ 90 mmHg and/or currently taking antihypertensive drugs were considered as being hypertensive individuals.

Diabetes was defined as having an FPG of ≥126 mg/dl and/or being on diabetes medication and/or if the diabetes was confirmed by a health practitioner. Diabetes awareness was considered based on the self-report of the participants about previous diagnosis of diabetes. The diabetic patients who received treatments with a FPG less than 126 mg/dl were considered as the control FPG group. The study design and protocol were approved by the Committee on Research Ethics at Kermanshah University of Medical Sciences (Code: KUMS.REC.1394.315).

### 2.3. Statistical Analysis

The prevalence of DM, the proportion of aware patients, the probability of receiving treatment, and control of plasma glucose were estimated with 95% confidence interval. Univariate and multivariate logistic regression analyses were performed to estimate the crude and adjusted odds ratios at a significance level of *p* ≤ 0.05.

## 3. Results

Initially, 10065 participants aged 35-65 years were recruited, 66 participants of which were excluded because they did not provide blood samples for FPG measurements. Approximately 52% of the participants were females, with a mean age of 47.33 ± 9.05 years. Of the total study population, 24.82% were illiterate, 60% lived in urban areas, and more than 90% were married.

### 3.1. Prevalence, Awareness, Treatment of Diabetes, and Glycemic Control

Based on the DM definition given above, the prevalence of diabetes was 8.19% (95% CI: 7.66-8.74). The mean ± standard deviation values of FPG were 97.06 ± 30.21, 97.14 ± 30.99, and 96.99 ± 29.49 in the whole included sample, male, and female, respectively. The corresponding values for the diabetic patients, subjects who were aware of their condition, under treatment participants, and those under glycemic control were 172.08 ± 62.57, 172.60 ± 65.19, 168.58 ± 69.21, and 105.77 ± 15.71, respectively. Among the diabetic patients, 74.97% were aware of their disease status, 56.04% were receiving treatment, and 18.3% were under glycemic level control. The presence of healthy diet was higher in DM patients than the majority of healthy participants (*p* < 0.01; Table 1).

### 3.2. Diabetes Risk Factors

The risk of DM was equal in male and female participants (*p* = 0.454) and was directly associated with age and reversely correlated with education (*p* < 0.01 and *p* < 0.01, respectively). The risk was significantly higher in the divorced, widowed, and urban residents than in the other participants (*p* < 0.01); however, there were no significant differences across the socioeconomic subgroups (*p* = 0.306). A higher BMI value was positively associated with a higher risk of DM, and participants with central obesity were more likely to suffer from DM than the others (*p* < 0.01). Although the risk of DM in the current smokers and never-smokers was similar, it was significantly higher in the ex-smokers and those suffering from both hypertension and dyslipidemia (*p* < 0.01). Also, the risk was significantly higher in participants with a positive family history of DM in their first-degree relatives and those with the least physical activity level than in the other participants (*p* < 0.01; Table 1). The results of our multiple regression analysis demonstrated that old age; living in urban areas; being obese or overweight; suffering from comorbidities including dyslipidemia, hypertension, or both of them; and having positive family history especially in the first-degree relatives were strongly associated with a higher risk of DM. Meanwhile, engagement in physical activity was a protective factor; however, consuming healthy diets was more prevalent in DM patients than in the general population (Table 2).

### 3.3. Associated Factors of DM Awareness

Higher awareness was observed in female subjects (*p* < 0.01). The awareness was positively associated with age (*p* < 0.005). DM awareness was not different significantly across people with different levels of education (*p* = 0.614), marital status, (*p* = 0.867), and socioeconomic status (*p* = 0.172); it was also the same in rural and urban residents (*p* = 0.403). The awareness was negatively associated with increasing BMI (*p* < 0.01). While the awareness was lower in people with central obesity, it was on the edge of significance (*p* = 0.056). The awareness was relatively higher in people who suffered from both dyslipidemia and hypertension and those with a positive family history in their first-degree relatives (*p* < 0.05). In addition, the awareness in the current smokers was significantly lower than ex-smokers or nonsmokers (*p* = 0.035), as well as in people with high physical activity (*p* < 0.05). Participants with a healthy diet were partially more aware, but it was not statistically significant (*p* = 0.503) (Table 1).

Results of the multiple regression model showed that awareness was more than two times higher in females (OR = 2.27, 95% CI: 1.5, 3.43). In addition, those aged ≥55 years were more likely to be aware of their conditions (OR = 1.82, 95% CI: 1.09, 3.04). People who suffered from dyslipidemia and hypertension simultaneously (OR = 2.38, 95% CI: 1.42, 4.01) and those with a positive family history in their first-degree relatives (OR = 2.07, 95% CI: 1.48, 2.90) showed higher awareness of DM. It was significantly lower by 42% in obese people compared to those with normal and underweight BMIs (OR = 0.42, 95% CI: 0.23, 0.74) (Table 2).

### 3.4. Associated Factors of Treatment

The probability of receiving treatment was the same for males and females (*p* = 0.456) in different age groups (*p* = 0.871) with different educational levels (*p* = 0.127). It was significantly lower in singles than the others (*p* < 0.017) and in those with higher BMI (*p* < 0.01). There were no statistically significant differences between urban and rural residents and across the socioeconomic subgroups. In addition, significant differences were not recorded in the probability of receiving treatment between participants with central obesity and normal subjects (*p* = 0.144), between people with/without comorbidities (*p* = 0.685), and between nonsmokers, current smokers, and ex-smokers (*p* = 0.495). Participants with a positive family history in the first-degree relatives were more likely to receive treatments; however, it was not statistically significant (*p* = 0.061). The probability of receiving treatment decreased with regular levels of physical activity, but it was not statistically significant (*p* = 0.55). Lastly, the probability of receiving treatment was the same in both patients with healthy and unhealthy diets (*p* = 0.447; see Table 1).

After adjusting for the effects of age, BMI, waist to hip ratio, comorbidities, family history, and physical activity in the multiple regression model, obese, married, and widowed subjects were more likely to receive treatments. The probability of receiving treatments was 65% lower in obese than normal weight participants (OR = 0.35, 95% CI: 0.20, 0.62). It was 10 and 9 times higher in married and widowed/divorced subjects, respectively (OR = 10.04 and 9.40), which were statistically significant (Table 2).

### 3.5. Associated Factors of Control

The probability of controlling the glycemic level was not statistically different in male and female participants (*p* = 0.558) or within different age groups (*p* = 0.513). However, it was significantly higher in people with 6-9 years of schooling (*p* < 0.05). The risk of the controlled glycemic level was similar for different categories of marital status (*p* = 0.194), residential areas (*p* = 0.531), socioeconomic status (*p* = 0.949), BMIs (*p* = 0.307), waist to hip ratios (*p* = 0.595), comorbidities (*p* = 0.146), smoking (*p* = 0.772), and family history of DM (*p* = 0.643), physical activity level (*p* = 0.772) and those who consumed healthy diets or not (*p* = 0.895) (Table 1). Based on the results of multiple regressions, we adjusted for the effect of education and comorbidities. Participants suffering from hypertension were more likely to have their plasma glucose controlled than those with no comorbidities (OR = 2.32, 95% CI: 1.17, 4.58). Compared to illiterate subjects, 6-9 years of schooling was associated with a higher chance of plasma glucose control (OR = 1.91; see Table 2).

## 4. Discussion

The prevalence of diabetes was 8.19% among a 9999 sample of people participating in a first cohort in a Kurdish settlement in the western part of Iran. Of all diabetic participants, around 75 percent were aware of their disease status, 75% of which were under the treatment while glycemic control was achieved in just 32.68% of the patients who were receiving the treatments. Based on the results, the DM prevalence was lower than the previous national estimates and recent subnational estimates. Esteghamati et al. [2] reported the prevalence of DM in Iran as 11.37% (95% CI: 9.86, 12.89). Prevalence of DM in a sample of 40- to 64-year-old participants in Shahroud was 12.3%, and in Yazd, among people 40 to 80 years old, reported as 24.5% [19, 20]. Yazdanpanah et al. [10] reported that around 15% of people in Ahvaz suffered from DM, which is higher than the prevalence reported in the present study. The prevalence of DM in here is close to those reported in China or Jamaica and lower than those in Ecuador, Kazakhstan, Portugal, and Turkey. These may be due to differences in age distribution of subjects, demographics, and lifestyle [9, 21–25].

Consistent with the results of previous studies [26, 27], BMI and age were directly associated and physical activity was conversely associated with the risk of DM. Similarly, the risk of DM herein was far higher in those suffering from concurrent hypertension and dyslipidemia [10, 22, 23]. Ex-smokers were at a higher risk of DM than the nonsmokers as was also reported elsewhere [22]. Positive family history in both immediate and second degree relatives was a reliable determinant and directly associated with a higher risk of diabetes [10].

In previous studies, we found that inverse associations were reported between consumption of healthy diet and the risk of DM [22, 28, 29]. Likewise, unhealthy diets may decrease the risk of DM, and a higher proportion of the diabetic patients were on healthy diets. This may be largely influenced by behavioral changes after diabetes since this association was not seen after assessment of newly diagnosed diabetic cases (data not shown). It seems that changing the behavior and tendency toward more healthy nutrition in diabetic patients can be the main reason for this relationship. Kim et al. [30] have recently reported that diabetic and prediabetic patients tend to eat less sugar, fat, and carbohydrates and more fruits. Few studies have explored the association; hence, further studies in the future are recommended on changing the lifestyle and moving toward healthier behaviors.

Comparing to the national estimates in 2011, the DM awareness was approximately 76%, which was slightly higher than the awareness level found in our study population. However, the level of awareness in this study was higher than that reported for Ahvaz (40.4%) [10], Kerman (70%) [31], and Isfahan (54%) [11] and for some other countries, such as China (52.5%) [22], Bangladesh (41.2%) [32], Malaysia (65.2%) [33], and Kazakhstan (72.3%) [25]. Also, it was lower than the results reported for Portugal (87.1%) [9], southern cone of Latin America (79.9%) [34], and Jamaica (75%) [21]. Consistent with the results of the other studies, awareness of DM was higher for females and older patients in our study, but it was lower in obese and overweight individuals [33]. Also, suffering from certain comorbidities increases the awareness of DM apparently due to higher probability of seeking care for the reasons other than diabetes such as hypertension or dyslipidemia [10, 22]. Also, having a diabetic patient among the first-degree family members increases the awareness of DM [10, 22].

In this study, approximately 75% of diabetic patients received treatment to manage their DM. The percentage of those receiving care for DM was still lower than that reported for Malaysia (87.5%) [33] and Portugal (79.7%) [9] but higher than those for Kazakhstan (65.6%) [25], southern cone of Latin America (58.8%) [34], China (48.1%) [22], and Bangladesh (36.9%) [32]. Based on the results of this and other studies, receiving treatment was highly associated with obesity in patients with DM [35].

Despite the considerable probability of the patients receiving treatment in this study, only 32.68% of them had their plasma glucose controlled, which was lower than those reported for Portugal (63.2%) [9] or southern cone of Latin America (46.2%) [34]. However, it was higher than Malaysia (21.8%) [33], Kazakhstan (27.7%) [25], China (19.1%) [22], and Bangladesh (14.2%) [32]. The results of previous studies emphasize attention to the effectiveness of DM treatment in rural and urban areas of Iran [36]. In this study, only one-third of the patients receiving treatment for DM had normal FPG. Suffering from hypertension alone or combined with dyslipidemia was associated with better plasma glucose control. This may be due to better adherence to the treatment among such patients. The effects of some variables on plasma glucose control were assessed in here while most of the patients were not able to predict it. Furthermore, it is highly recommended to research on the identification of main predictors of plasma glucose control, particularly biological factors.

Based on our previous report of hypertension status in the RaNCD cohort study (unpublished data), while the prevalence of hypertension was more than diabetes (15% vs. 7%), the awareness, treatment, and control of hypertension were 80.7%, 73.2%, and 53.3%, respectively. A comparison of the status of the two chronic conditions revealed that despite the similar treatment proportion (75% vs. 73%), control of hypertension was better achieved than that for diabetes (53% vs. 33%). Data on nonpharmacological treatments were not collected in the current study. In fact, differences between hypertension and diabetes need further investigations to explore patients' adherence to the treatment and the guidelines prescribed by physicians.

Our study had certain limitations, the most important of which was to rely heavily on prevalence data, while the effect of survival bias could not be ignored. We did not differentiate between type I and II DM although it was likely that our adult patients were more likely to have diabetes type II. In addition, the authors did not differentiate between prediabetic participants receiving advices on diet and/or physical activity to decrease the risk of diabetes and those who actually had diabetes. Furthermore, based on our protocol in the PERSIAN cohort study, we did not measure HbA1c because of financial limitations. In fact, HbA1c is more precise to assess diabetes control than fasting plasma glucose as it shows the control of diabetes over a longer period of time.

On the other hand, prevalence of comorbidities in diabetic patients is a matter of attention that was not fully assessed in the present study. The prevalence of diabetes among those with hypertension, dyslipidemia, or both was 11.57%, 9.49%, and 23.4%, respectively, which was much greater than the normal population. However, the prevalence of hypertension in the diabetic patients was around 34%, and in nondiabetic participants, it was 14% (results were not presented). In terms of CVD history, 69.2% of people with diabetes and history of CVD had hypertension. The corresponding value for those that suffered from diabetes but without history of CVD was 20.3%. Although our values are lower than what have been reported by Rabizadeh et al. in 2019, it can be said that they are comparable to some extent [37]. Rabizadeh et al. reported the history of hypertension in diabetic patients without coronary artery disease and diabetic patients with the history of coronary artery disease as 38.4% and 62.8%, respectively [37]. Considering the younger age of diabetic patients in our sample (mean of age: 52.66 vs. 55.68 years) plus the possible dilution effect of having both prevalent cases of diabetes and newly diagnosed patients (which might not have developed the hypertension yet), such differences are understandable [37]. So, it is highly recommended to assess the comorbidities in diabetic patients in future researches.

Roughly, nine out of 1000 people in this Iranian Kurdish subgroup are suffering from diabetes. Regarding its cost and related complications, it is highly recommended to adopt health measures to decrease the detrimental effects. Although the awareness and probability of receiving treatment were fairly acceptable, control of the plasma glucose in patients receiving treatment needs more attention. More research is necessary on the potential factors contributing to the glycemic control of DM to identify high-risk subgroups. It is, therefore, highly recommended to establish national estimates for the assessment of diabetes awareness, probability of treatment, control of plasma glucose levels, and the effectiveness of treatments across the country.

## Figures and Tables

**Table 1:** Prevalence, awareness, treatment, and glycemic control of diabetes by other important factors in the RaNCD cohort study.

Variables	*N*	Diabetes prevalence (95% CI)	Awareness (95% CI)	Treatment (95% CI)	Glycemic control (95% CI)
*Total*	*9999*	*8.19 (7.67, 8.74)*	*74.97 (71.88, 77.82)*	*74.75 (71.16, 78.04)*	*32.68 (28.53, 37.12)*
*Gender*					
Males	4740	7.97 (7.24, 8.78)	79.05 (64.19, 73.52)	76.24 (70.68, 81.03)	33.16 (26.94, 40.03)
Females	5259	8.38 (7.77, 9.16)	80.04 (76.04, 83.52)	73.65 (68.79, 78.01)	32.31 (26.88, 38.26)
*p* value		0.454	<0.0001	0.456	0.846
*Age group (year)*					
35-44	3544	3.07 (2.55, 3.70)	66.97 (57.57, 75.18)	72.60 (61.19, 81.66)	39.62 (27.32, 53.39)
45-54	3593	8.85 (7.96, 9.82)	71.39 (66.15, 76.09)	74.45 (68.35, 79.72)	31.36 (24.78, 38.78)
55 and more	2861	13.70 (12.49, 15.01)	80.10 (75.84, 83.77)	75.48 (70.39, 79.93)	32.07 (26.41, 38.30)
*p* value		<0.0001	0.003	0.871	0.513
*Marital status*					
Single	420	2.38 (1.28, 4.37)	71.53 (35.88, 90.68)	28.57 (6.35, 70.23)	50.00 (1.92, 98.07)
Married	9017	8.34 (7.79, 8.93)	74.87 (71.63, 77.84)	75.49 (71.75, 78.87)	33.65 (29.29, 38.29)
Widow/divorced/other	561	10.16 (7.92, 12.95)	77.19 (64.43, 86.35)	72.73 (57.65, 83.93)	18.75 (8.54, 36.31)
*p* value		<0.0001	0.867	0.017	0.194
*Education (year)*					
Illiterate	2482	10.87 (9.71, 12.16)	75.92 (70.45, 80.67)	77.56 (71.31, 82.78)	34.59 (27.57, 42.35)
1-5 years	3821	8.29 (7.46, 9.21)	76.34 (71.33, 80.71)	70.66 (64.59, 76.07)	29.24 (22.88, 36.53)
6-9 years	1657	7.18 (6.03, 8.53)	70.58 (61.75, 78.11)	82.14 (72.38, 88.98)	46.37 (34.91, 58.24)
10 and more	2038	5.54 (4.63, 6.63)	73.45 (64.51, 80.81)	72.29 (61.64, 80.90)	21.67 (12.94, 33.98)
*p* value		<0.0001	0.614	0.127	0.016
*Residential areas*					
Urban	5909	9.12 (8.41, 9.88)	75.88 (72.07, 79.31)	74.08 (69.60, 78.11)	33.66 (28.54, 39.19)
Rural	4090	6.84 (6.11, 7.66)	73.21 (67.70, 78.09)	76.09 (69.75, 81.46)	30.77 (23.99, 38.49)
*p* value		<0.0001	0.403	0.588	0.531
*Economic status*					
Poorest	1993	7.58 (6.49, 8.82)	75.50 (67.97, 81.73)	74.56 (65.73, 81.75)	34.12 (24.78, 44.87)
2nd poorest	1991	8.79 (7.62, 10.11)	73.14 (66.06, 79.21)	72.66 (64.25, 79.71)	31.18 (22.55, 41.35)
Middle	1989	8.95 (7.78, 10.28)	80.90 (74.42, 86.04)	75.69 (67.98, 82.03)	34.86 (26.46, 44.32)
2nd richest	1992	8.03 (6.92, 9.31)	69.37 (61.77, 76.05)	76.58 (67.75, 83.57)	34.12 (24.78, 44.87)
Richest	1975	7.49 (6.41, 8.74)	75.67 (68.08, 81.94)	74.11 (65.16, 81.41)	30.12 (21.17, 40.89)
*p* value		0.306	0.172	0.964	0.949
*BMI (9956)*					
≤18.4: underweight	2937	5.17 (4.43, 6.04)	82.24 (75.30, 87.55)	84.00 (76.44, 89.46)	26.67 (19.04, 35.99)
18.5-24.99: normal	4311	8.30 (7.52, 9.17)	76.81 (72.15, 80.91)	76.00 (70.57, 80.69)	34.93 (28.74, 41.67)
25.0 and more: overweight	2678	11.02 (9.88, 12.26)	68.47 (62.93, 73.54)	66.34 (59.51, 72.54)	34.33 (26.74, 42.81)
*p* value		<0.0001	0.003	0.001	0.307
*WHR (n* = 9962)					
Normal	1752	4.51 (3.63, 5.59)	83.54 (73.59, 90.24)	81.82 (70.53, 89.43)	29.63 (18.91, 43.19)
Central obesity	8174	8.89 (8.28, 9.52)	73.83 (70.50, 76.91)	73.51 (69.59, 77.08)	33.25 (28.75, 38.07)
*p* value		<0.0001	0.059	0.144	0.595
*Smoking habit*					
Nonsmoker	7976	7.85 (7.28, 8.46)	76.36 (72.86, 79.53)	75.52 (71.45, 79.18)	32.13 (27.49, 37.15)
Smoker	1169	7.53 (6.15, 9.19)	63.63 (53.05, 73.05)	75.00 (61.19, 84.68)	33.33 (20.68, 48.95)
Ex-smoker	826	12.47 (10.38, 14.90)	66.47 (66.47, 83.07)	69.23 (58.07, 78.51)	37.04 (25.17, 50.71)
*p* value		<0.0001	0.035	0.495	0.772
*Daily physical activity (METs)*					
24-36.5 = low	2748	9.82 (8.77, 10.99)	76.67 (71.23, 81.34)	77.29 (71.06, 82.51)	31.25 (24.51, 38.88)
36.6-44.9 = moderate	5137	8.27 (7.55, 9.06)	76.47 (72.19, 80.27)	73.84 (68.78, 78.35)	34.17 (28.42, 40.42)
≥45 = vigorous	2107	5.88 (4.96, 6.97)	66.13 (57.32, 73.94)	71.95 (61.21, 80.65)	30.51 (20.04, 43.46)
*p* value		<0.0001	0.048	0.551	0.772
*Family history of hypertension (n* = 10022)					
None	6574	5.62 (5.09, 6.21)	68.11 (63.17, 72.67)	74.60 (68.84, 79.61)	32.98 (26.60, 40.05)
Second degree	839	7.64 (6.12, 9.76)	75.38 (63.40, 84.41)	61.22 (46.88, 73.86)	40.00 (24.04, 58.40)
First degree	2586	14.85 (13.53, 16.27)	81.51 (77.29, 85.09)	76.99 (71.99, 81.34)	31.53 (25.96, 37.70)
*p* value		<0.0001	<0.0001	0.061	0.643
*Comorbidities*					
None	4835	4.05 (3.53, 4.65)	67.86 (60.97, 74.04)	72.93 (64.71, 79.83)	25.77 (17.99, 35.45)
Hypertension	726	11.57 (9.44, 14.11)	77.38 (67.15, 85.13)	78.46 (66.72, 86.87)	45.09 (31.97, 58.95)
Dyslipidemia	3592	9.49 (8.58, 10.49)	73.31 (68.35, 77.75)	73.20 (67.34, 78.34)	31.69 (25.33, 38.82)
Both	846	23.40 (20.67, 26.37)	83.84 (78.01, 88.35)	77.11 (70.07, 82.89)	34.37 (26.62, 43.06)
*p* value		<0.0001	0.002	0.679	0.115
*Healthy eating index*					
	5409	6.93 (6.28, 7.64)	73.87 (69.17, 78.07)	73.28 (67.74, 78.18)	33.00 (26.86, 39.79)
	4590	9.67 (8.85, 10.56)	75.90 (71.69, 79.66)	75.96 (71.09, 80.24)	32.42 (26.95, 38.42)
*p* value		<0.0001	0.503	0.447	0.895

**Table 2:** Predictor factors of prevalence, awareness, treatment, and glycemic control of the diabetes RaNCD cohort study based on the results of the univariate and multivariate models.

Variables			Prevalence		Awareness		Treatment		Controlled	
			Crude OR (95% CI)	Adjusted OR (95% CI)	Crude OR (95% CI)	Adjusted OR (95% CI)	Crude OR (95% CI)	Adjusted OR (95% CI)	Crude OR (95% CI)	Adjusted OR (95% CI)
Gender	(Ref: male)		1.06 (0.92, 1.22)		1.80 (1.31, 2.48)	2.27 (1.5, 3.43)	0.88 (0.61, 1.27)		0.93 (0.64, 1.34)	

Age	(Ref: 35-44)	45-54	3.06 (2.45, 3.83)	2.74 (2.17, 3.47)	1.24 (0.78, 1.97)	1.38 (0.84, 2.26)	1.10 (0.61, 2.00)		0.76 (0.42, 1.37)	
		54 and more	5.01 (4.03, 6.23)	4.02 (3.09, 5.21)	1.99 (1.25, 3.18)	1.82 (1.09, 3.04)	1.17 (0.66, 2.07)		0.8 (0.45, 1.4)	

Marital status	(Ref: single)	Married	3.74 (1.99, 7.02)	1.57 (0.82, 3.02)	1.28 (0.33, 4.99)		7.7 (1.48, 40.14)	10.04 (1.87, 54.14)	2.05 (0.25, 17.12)	
		Widow/divorced/other	4.64 (2.34, 9.20)	1.33 (0.65, 2.73)	1.46 (0.33, 6.42)		6.67 (1.14, 39.1)	9.40 (1.54, 57.37)	0.95 (0.1, 9.32)	

Education	(Ref: illiterate)	1-5 years	0.75 (0.63, 0.88)	1.04 (0.85, 1.26)	1.03 (0.70, 1.50)		0.70 (0.46, 1.08)		0.72 (0.46, 1.11)	0.75 (0.49, 1.17)
		6-9 years	0.64 (0.51, 0.80)	1.07 (0.83, 1.39)	0.77 (0.47, 1.24)		1.34 (0.70, 2.55)		1.68 (0.99, 2.88)	1.91 (1.10, 3.34)
		10 and more	0.49 (0.39, 0.61)	0.86 (0.65, 1.14)	0.88 (0.54, 1.45)		0.76 (0.43, 1.36)		0.51 (0.26, 0.99)	0.58 (0.30, 1.14)

Residential area	(Ref: urban)		0.74 (0.64, 0.86)	0.89 (0.75, 1.05)	0.87 (0.63, 1.21)		1.12 (0.76, 1.65)		0.93 (0.63, 1.37)	

Economic status	(Ref: poorest)	2nd poorest	1.18 (0.94, 1.48)		0.89 (0.54, 1.46)		0.91 (0.52, 1.61)		0.86 (0.48, 1.55)	
		Middle	1.20 (0.96, 1.51)		1.38 (0.82, 2.33)		1.07 (0.61, 1.88)		1.06 (0.6, 1.85)	
		2nd richest	1.07 (0.85, 1.35)		0.74 (0.45, 1.22)		1.12 (0.61, 2.06)		1.04 (0.58, 1.89)	
		Richest	0.99 (0.79, 1.26)		1.01 (0.60, 1.72)		0.98 (0.54, 1.78)		0.85 (0.46, 1.56)	

BMI	(Ref: ≤18.4)	18.5-24.9	1.66 (1.37, 2.02)	1.21 (0.97, 1.52)	0.72 (0.45, 1.17)	0.83 (0.47, 1.46)	0.61 (0.35, 1.05)	0.57 (0.33, 1.00)	1.26 (0.77, 2.07)	
		25.0 and more	2.27 (1.86, 2.78)	1.43 (1.12, 1.82)	0.47 (0.29, 0.77)	0.42 (0.23, 0.74)	0.38 (0.22, 0.66)	0.35 (0.2, 0.62)	1.03 (0.6, 1.75)	

WHR	(Ref: normal)		2.07 (1.63, 2.62)	1.29 (0.97, 1.71)	0.56 (0.30, 1.04)	0.51 (0.24, 1.07)	0.62 (0.33, 1.19)		1.02 (0.56, 1.84)	

Smoking cigarette	(Ref: nonsmoker)	Current	0.96 (0.76, 1.21)	1.03 (0.80, 1.33)	0.55 (0.34, 0.87)	0.71 (0.42, 1.22)	0.98 (0.52, 1.85)		1.05 (0.55, 1.98)	
		Ex-smoker	1.68 (1.34, 2.09)	1.30 (1.02, 1.65)	0.97 (0.60, 1.58)	1.23 (0.71, 2.12)	0.73 (0.44, 1.24)		1.08 (0.63, 1.87)	

Physical activity Daily MET	(Ref: 24–36.5)	36.6-44.9	0.83 (0.71, 0.98)	0.94 (0.79, 1.12)	0.99 (0.69, 1.42)	0.82 (0.55, 1.22)	0.83 (0.56, 1.25)		1.06 (0.71, 1.59)	
		≥45	0.58 (0.47, 0.72)	0.76 (0.59, 0.96)	0.60 (0.38, 0.95)	0.67 (0.40, 1.11)	0.76 (0.43, 1.35)		0.89 (0.48, 1.63)	

Family history of hypertension	(Ref: no)	Second degree	1.41 (1.08, 1.86)	1.40 (1.05, 1.85)	1.44 (0.79, 2.63)	1.55 (0.82, 2.91)	0.54 (0.29, 1.03)		1.00 (0.49, 2.03)	
		First degree	2.93 (2.52, 3.41)	3.13 (2.67, 3.68)	2.07 (1.48, 2.90)	2.17 (1.51, 3.10)	1.14 (0.78, 1.68)		0.99 (0.67, 1.45)	

Comorbidity	Ref: none	Hypertension	3.10 (2.37, 4.06)	2.00 (1.51, 2.66)	1.63 (0.90, 2.94)	1.47 (0.78, 2.76)	1.36 (0.67, 2.74)		2.37 (1.22, 4.62)	2.32 (1.17, 4.58)
		Dyslipidemia	2.49 (2.08, 2.98)	2.22 (1.84, 2.68)	1.31 (0.89, 1.92)	1.50 (0.99, 2.26)	1.02 (0.64, 1.63)		1.31 (0.78, 2.21)	1.25 (0.74, 2.13)
		Both	7.24 (5.84, 8.96)	4.43 (3.51, 5.59)	2.46 (1.52, 3.99)	2.38 (1.42, 4.01)	1.26 (0.74, 2.12)		1.56 (0.90, 2.72)	1.64 (0.93, 2.9)

Healthy diet	Ref: yes	No	0.70 (0.61, 0.81)	0.77 (0.66, 0.9)	0.90 (0.66, 1.24)		0.87 (0.61, 1.26)		1.02 (0.56, 1.84)	—

## Data Availability

The data used to support the findings of the present study are available from the corresponding author upon request.
